# Subclinical microvascular changes in ANCA-vasculitides: the role of optical coherence tomography angiography and nailfold capillaroscopy in the detection of disease-related damage

**DOI:** 10.1186/s13023-023-02782-7

**Published:** 2023-07-10

**Authors:** P Triggianese, A D’Antonio, C Nesi, B Kroegler, M Di Marino, P Conigliaro, S Modica, E Greco, C Nucci, A Bergamini, MS Chimenti, M Cesareo

**Affiliations:** 1 Rheumatology, Allergology and Clinical Immunology, Department of “Medicina dei Sistemi”, University of Rome Tor Vergata, Rome, 00133 Italy; 2 Ophthalmology Unit, Department of Experimental Medicine, University of Rome Tor Vergata, Rome, 00133 Italy

**Keywords:** Anti-neutrophil cytoplasm autoantibodies, Damage, Optical coherence tomography angiography, Retina, Vasculitides

## Abstract

**Background:**

Both cardiovascular and complement-mediated disorders might lead to microvascular damages in anti-neutrophil cytoplasm autoantibodies (ANCA)-associated vasculitides (AAV). We aimed at investigating, for the first time, subclinical microvascular abnormalities with non-invasive techniques in AAV patients by analyzing both retinal and nailfold capillary changes. Retinal plexi were investigated using optical coherence tomography angiography (OCT-A), while nailfold capillary changes by video-capillaroscopy (NVC). Potential correlations between microvessels’ abnormalities and disease damage were also explored.

**Methods:**

An observational study was conducted on consecutive patients who met the inclusion criteria of defined diagnosis of eosinophilic granulomatosis with polyangiitis (EGPA), granulomatosis with polyangiitis (GPA), and microscopic polyangiitis (MPA), age ≥ 18 ≤ 75 yrs, and no ophthalmological disorders. Disease activity was assessed by Birmingham Vasculitis Activity Score (BVAS), damage by Vasculitis Damage Index (VDI), and poorer prognosis by the Five Factor Score (FFS). Quantitative analysis of vessel density (VD) was performed by OCT-A in both superficial and deep capillary plexi. Figures and detailed analysis from NVC were performed for all subjects in the study.

**Results:**

Included AAV patients (n = 23) were compared with 20 age/sex-matched healthy controls (HC). Retinal VD in superficial whole and parafoveal plexi resulted significantly decreased in AAV compared to HC (P = 0.02 and P = 0.01, respectively). Furthermore, deep whole and parafoveal vessel density was strongly reduced in AAV than HC (P ≤ 0.0001 for both). In AAV patients, significant inverse correlations occurred between VDI and OCTA-VD in both superficial (parafoveal, P = 0.03) and deep plexi (whole, P = 0.003, and parafoveal P = 0.02). Non-specific NVC pattern abnormalities occurred in 82% of AAV patients with a similar prevalence (75%) in HC. In AAV, common abnormalities were edema and tortuosity in a comparable distribution with HC. Correlations between NVC changes and OCT-A abnormalities have not been described.

**Conclusion:**

Subclinical microvascular retinal changes occur in patients with AAV and correlate with the disease-related damage. In this context, the OCT-A can represent a useful tool in the early detection of vascular damage. AAV patients present microvascular abnormalities at NVC, whose clinical relevance requires further studies.

## Background

Anti-neutrophil cytoplasm autoantibodies (ANCA)-associated vasculitides (AAV) are rare diseases characterized by a blood vessel inflammation resulting in organ dysfunctions [1–3]. Granulomatosis with polyangiitis (GPA), previously known as Wegener’s granulomatosis, is the most common AAV and it is characterized by granulomatous and necrotizing inflammation that usually involves the upper and lower respiratory tract, and necrotizing vasculitis of small to medium vessels [4]. Also, the Eosinophilic Granulomatosis with Polyangiitis (EGPA) - formerly known as Churg-Strauss Syndrome (CSS) - is characterized by granulomatous and necrotizing vasculitis affecting small-to-medium sized vessels, but it is peculiarly typified by an eosinophil rich inflammation associated with severe bronchial asthma, and hyper eosinophilia [5]. Microscopic polyangiitis (MPA) is the small vessels necrotizing vasculitis without both the granulomatous inflammation and the relevant immune deposits [6]. A third of AVV patients shows ANCA, mainly p-ANCA that recognize, in 80–90% of cases, the myeloperoxidase (MPO-ANCA) with a perinuclear/nuclear staining using indirect immunofluorescence on ethanol-fixed neutrophils; p-ANCA are preferentially associated with MPA [4–6]. However, c-ANCA are mainly associated with GPA and are directed against the proteinase 3 (PR3-ANCA) showing a diffuse cytoplasmic staining [7]. EGPA patients with MPO-ANCA have been reported to show a more “vasculitic phenotype,” with the respect to the ANCA negative patients who often present peripheral neuropathy, palpable purpura, glomerulonephritis and, rarely, alveolar hemorrhage [8]. EGPA is believed to have a better prognosis than other types of AAV [9] though cardiac involvement is an independent risk factor of mortality [10]. In AAV, Birmingham Vasculitis Activity Score (BVAS) is used to define patients’ disease activity [11] while the damage can be evaluated by using the Vasculitis Damage Index (VDI), which is a comprehensive and validated clinical checklist that records the accumulation of damage from the disease onset [12]. An AAV prognosis measure is the Five Factor Score (FFS), which has significant prognostic value and correlates with the presence of five clinical presentations (renal impairment, proteinuria, and involvement of the cardiovascular, gastrointestinal, and central nervous systems) [11]. Among the organs and systems potentially involved in AAV, the eye can be affected often in GPA, with changes in orbital tissues, conjunctiva, eyelids, and cornea [13, 14]. Posterior segment is less commonly involved in AAV, and abnormalities in retinal vessels and/or choroidal circulation have been rarely documented [14, 15]. Only few case reports documented occlusive vasculitides in retinal network, particularly in ANCA negative AAV patients [16–28].

The prevalence of microvascular retinal changes, particularly in a pre-symptomatic phase, has not been thoroughly investigated in AAV patients. Furthermore, a routine screening of retinal abnormalities is not recommended for individuals with AAV excepted for the detection of possible toxic damages related to treatments [15].

Evidence from the literature supports the role of the optical coherence tomography angiography (OCT-A) as a non-invasive diagnostic tool for the early detection of subclinical retinopathy in systemic autoimmune diseases [29]. Accordingly, we recently documented, for the first time, subclinical abnormalities in retinal microvascular network in patients with Systemic Lupus Erythematosus (SLE) by using OCT-A [30–33].

In recent years, an increasing focus has emerged on the usefulness of the non-invasive microvascular examination also at the nailfold bed in the context of several systemic diseases. Specifically, nail bed capillaroscopy is to evaluate patients with suspicion of systemic sclerosis (mainly), mixed connective tissue disease, and other autoimmune diseases [34]. It i salso used in non-autoimmune disorders [35–38]. The nailfold capillary evaluation by using videocapillaroscopy (NVC) has been interestingly performed in patients with diabetes mellitus, arterial hypertension, and in subjects with ophthalmologic disorders such as glaucoma and chorioretinitis [35–37]. Furthermore, recent findings documented NVC-microangiopathic patterns in AAV patients, with a possible correlation with disease activity [38].

We aimed at exploring for the first time subclinical microvascular changes in AAV patients by both OCT-A at retinal level and NVC. Furthermore, potential correlations between microvascular findings and disease activity and damage have been analyzed.

## Methods

A monocentric cross-sectional observational study, from 1 September 2020 to 31 October 2021, was conducted on patients with established AAV recruited from the tertiary care center of Rheumatology Unit, Tor Vergata University Hospital in Rome (Italy).

Inclusion criteria were: (1) a diagnosis of EGPA, GPA, and MPA [3, 39]; (2) age ≥ 18 and ≤ 75 years; (3) intraocular pressure (IOP) < 21 mmHg on diurnal testing with measurements using Goldmann applanation tonometry; (4) best-corrected visual acuity (BCVA) ≥ 0.5 logMAR; (5) spherical equivalent refractive error between − 6.0 and + 4.0 diopters [30–32]. Exclusion criteria were: (1) established primary ocular diseases including glaucoma; (2) systemic disorders with known retinal involvement such as diabetes, severe renal dysfunctions, and other autoimmune systemic diseases (current and past medical history); (3) neoplasia; (4) pregnancy or lactation; (5) systemic treatments affecting retinal function [30–32, 40].

Among 47 consecutive AAV patients referring to the Rheumatology Unit, during the considered time interval, 23 patients fulfilled inclusion criteria and were compared with 20 healthy controls (HC). Clinical data, therapies, and accumulated damage were registered.

Clinical records included age at the disease onset/diagnosis, disease duration, concomitant disorders, and therapies. From all the patients in the study, serum levels of complement components C3 and C4, determination of MPO/PR3-ANCA, total Immunoglobulin (Ig)E, anti-nuclear antibodies (ANA) titer, rheumatoid factor (RF) were obtained. Levels of C3 and C4 were measured using nephelometric assays (normal values 90–180 mg/dL and 10–40 mg/dL for C3 and C4, respectively), while PR3 and MPO were determined with Chemiluminescent Immunoassay (CLIA, normal values < 20 U for both). ANA detection was conducted using IFA performed with HEp-2 cells (negative at the 1:80 dilution). The quantitative measurement of IgE was obtained by immunoturbidimetric assay (normal values < 90 IU/ml). Serum levels of glucose, creatinine, and 24-h proteinuria were also added to the panel to confirm glycemic homeostasis and renal function: normal values were 70–99 mg/dL for glucose, 0.7–1.2 mg/dL for creatinine, and < 300 mg/24 h for 24-h proteinuria.

AAV disease activity was assessed by expert rheumatologists by using BVAS, damage by VDI, and poorer prognosis by the FFS, in accordance with a good clinical practice [11, 12, 41, 42].

Nailfold vessel examinations were performed at the Rheumatology Unit by expert rheumatologists by using the NVC (Inspectis Digital Capillaroscope Light CAP-1). The following morphological and dynamic parameters were evaluated: capillary distribution (homogeneous vs. nonhomogeneous distribution of capillaries arranged in parallel to the distal row of the nail fold), capillary morphology (presence vs. absence of tortuous capillaries), capillary diameter (dilated capillaries, > 20 μm; capillary ectasia, 30–50 μm; megacapillaries, > 50 μm), capillary density (abnormal if number of capillary loops < 7 per linear mm), microhemorrhages (presence vs. absence), neoangiogenesis (presence vs. absence), edema (presence vs. absence), sub-papillary venous plexus (visible vs. non visible), flow (normal, granular, slow).

All subjects underwent ophthalmological evaluation at the Ophthalmology Unit of the Tor Vergata University Hospital in Rome (Italy). The best-corrected visual acuity (BCVA) was measured using a standard LogMAR eye chart according to the Early Treatment of Diabetic Retinopathy Study (ETDRS) protocol [43]. The IOP was measured by using Goldmann applanation tonometry [44–46]. Both eyes of each participant were examined with a 6 × 6 mm scanning protocol of the macula area using the Avanti Angiovue OCT-A (Optovue XR Avanti, Fremont, CA, USA). The vessel density (VD) was then calculated using the instrument’s built-in software. VD of both the superficial and deep plexi of the whole image, foveal, and parafoveal zone was recorded; foveal avascular zone (FAZ) area has been measured [30, 31, 44–46]. Exclusion criteria for a poor image quality according to specific criteria including low-quality index (< 7), presence of blink artifacts, motion or doubling artifacts caused by poor fixation, and media opacities obscuring the view of the vasculature [44–46]. All OCT-A measurements were performed at the same time of the day in both patients and controls and by the same expert ophthalmologist. Measures of the retinal thickness, both the foveal (FT) and the parafoveal thickness (PFT), obtained by using OCT-scans, were also registered, for completeness [32].

The control group consisted of 20 HC who were age/-sex and refractive index/-BCVA matched with AAV patients. Both eyes of each control were evaluated.

The study described has been carried out in accordance with The Code of Ethics of the World Medical Association (Declaration of Helsinki) for experiments involving humans (updated 2008). Informed consent was obtained from all subjects and the study was approved by the scientific ethic committee of the Tor Vergata University Hospital in Rome (Italy).

### Statistical analysis

D’Agostino and Pearson omnibus test was used to test the normality of data. Mean and standard deviation (SD) expressed normally distributed variables. Non-normally distributed variables were analyzed using median with percentile ranges. Categorical variables were presented with absolute frequencies and percentages. Continuous variables were compared using the parametric unpaired T test or the nonparametric Mann-Whitney U test when appropriate. Categorical variables were compared using the Chi-squared test or Fisher’ exact test when appropriate. The significance of any correlation was determined by Pearson’s correlation test. P values < 0.05 were considered significant. All statistical analyses were performed using GraphPad Prism version 9 (GraphPad software).

## Results

### Study population

The AAV cohort included 23 patients comprising EGPA and MPA in a similar prevalence (39%, both) while GPA represented minor cases (Table I). ANCA have been revealed in almost 70% of patients, mainly p-ANCA. None of patients was ANA positive. The mean age at AAV diagnosis was 60.9 ± 8.7 years while time from diagnosis to last follow-up was 9.6 ± 9.1 years with 25% of the cohort had one or more relapse. None of patients had reduced C3 and/or C4 at the time of the study nor significant 24-h proteinuria. Lung involvement represented the prevalent clinical finding followed by ear-nose-throat (ENT) and peripheral nervous system (PNS, Table I). Rare cases of gut and heart complications have been documented. No differences in clinical manifestations were detected between ANCA-positive and ANCA-negative patients. At study visit, only 4 patients (2%) had been off all therapy for more than 2 years during their follow-up.

A total of 46 eyes from AAV patients were analyzed. The BCVA values of AAV patients were within the normal range in each eye (0.01 ± 0.1, for both) and similar to those in HC (0.013 ± 0.03, for both). Furthermore, IOP in AAV patients (16.5 ± 3 right eyes, 16.7 ± 2.9 left eyes) were similar to IOP in HC (16 ± 3 both eyes).

### Retinal vessel density by OCTA

**Fig. 1:**
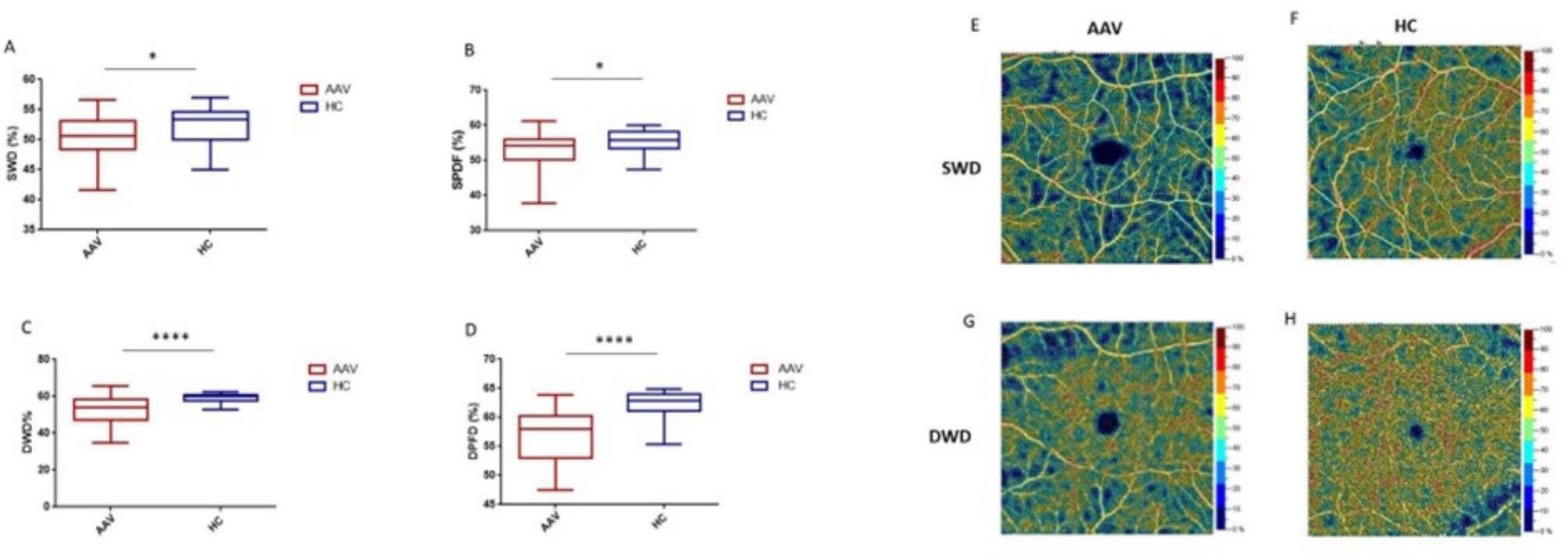
Superficial and deep retinal vessel density by optical coherence tomography angiography (OCT-A). Vessel density measures from patients (AAV) and controls (HC): in panel A, superficial whole density (SWD); in panel B, superficial parafoveal density (SPFD); in panel C, deep whole density (DWD); in panel D, deep parafoveal density (DPFD). Representative scans from OCT-A: in panels E and G, scans from a patient with Anti-neutrophil cytoplasm autoantibodies (ANCA)-vasculitides (AAV); in panels F and H, scans from a HC. Continuous variables were compared using the parametric unpaired T test (* p < 0.05, **** p < 0.0001)

Retinal vessel density (VD) in superficial whole (SWD) and superficial parafoveal vascular plexi (SPFD) were significantly decreased in AAV patients compared to HC (P = 0.02 and P = 0.01, respectively, Fig. 1A-B, Table II). Furthermore, deep whole (DWD) and deep parafoveal vessel density (DPFD) were strongly reduced in AAV patients than HC (P < 0.0001 for both, Fig. 1C-D, Table II). Representative scans from an AAV patient and a HC were reported in Fig. 1 (E-G from a patient and F-H from a HC). No significant difference in foveal vascular density occurred between AAV and HC, in both deep and superficial scans (Table II).

**Fig. 2:**
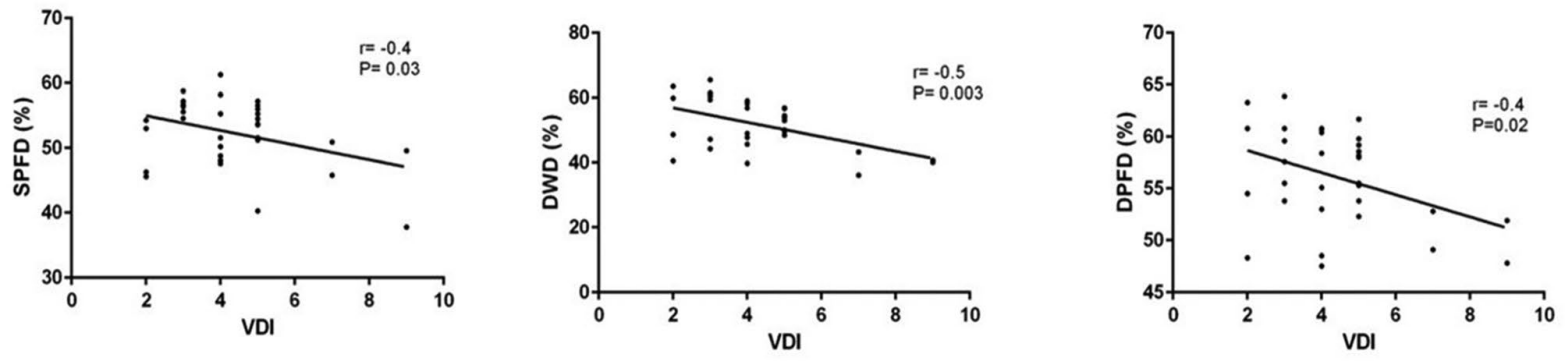
Correlations between Vasculitis Damage Index and Retinal Vessel Density. Correlations between Vasculitis Damage Index (VDI) and superficial parafoveal density (SPFD) in panel A, deep whole density (DWD) in panel B, and deep parafoveal density (DPFD) in panel C. The significance of any correlation was determined by Pearson’s correlation test

In AAV patients, significant inverse correlations emerged between VDI and SPFD (Pearson’s r -0.4, P = 0.03, Fig. 2A), DWD (Pearson’s r -0.5, P = 0.003, Fig. 2B), and DPFD (Pearson’s r -0.4, P = 0.02, Fig. 2C). Moreover, BVAS correlated directly with VDI (Pearson’s r -0.6, P = 0.001) and FFS (Pearson’s r -0.4, P = 0.01). Accordingly, BVAS was inversely correlated with disease duration (Pearson’s r -0.4, P = 0.01) and directly with VDI (Pearson’s r -0.6, P = 0.0003).

No correlations resulted between capillary density and complement components nor with age, age at the onset, disease duration, and diagnostic delay.

There were no differences in FAZ areas between the AAV patients and HC (Table II). In addition, retinal thickness measured by OCT was similar between AAV patients and HC (Table III).

### Nailfold evaluation with NVC

NCV investigation documented non-specific pattern abnormalities in 82.6% (n = 19/23) of AAV patients, with no differences from HC (75%, n = 15/20). Analyzing the type of alterations detected, almost all the cohort of patients showed pericapillary edema (69.6%, n = 16/23) and tortuosity (65.2%, n = 15/23). Similar prevalences resulted in HC (edema 60%, n = 12/20; tortuosity (65%, n = 13/20). Dilated capillaries were detectable in 17.4% (n = 4/23) of AAV and in 10% (n = 2/20) of HC. Rare cases of microhemorrhages (8.7%) and ectasias (8.7%) were recorded only in AAV cohort. No cases of megacapillaries, empty dermal papillae, and/or neoangiogenesis emerged in either group.

Functionally, half of the AAV population had slow flow (47.8%, n = 11/23), while a quarter of these had a granular flow (26%, n = 6/23). A similar distribution occurred in HC (slow flow in 35%, n = 7/20; granular flow in 20%, n = 4/20). Significant correlations between NVC changes and OCT-A abnormalities were not documented, nor between NCV findings and disease activity.

## Discussion

We documented for the first time subclinical microvascular changes in retinal vascular network from AAV patients and described significant correlations between vessel density abnormalities and AAV-disease damage.

As known, systemic vasculitis can present with a variety of clinical features, also including ocular changes [47–49]. In AAV, the increased morbidity and mortality is related to the multi-organ involvement secondary to inflammation and necrosis of the small blood vessels: the early detection of organ damages is, thus, the key challenge in the management of these diseases [14, 15, 26, 49]. However, the detection of ocular involvement in AAV, unless dramatic, often recognizes a relevant diagnostic delay that leads to a worse prognosis for patients [50]. Our data support the utility of the OCT-A as a non-invasive tool to the early detection of retinal vascular abnormalities in AAV patients in whom both the cardiovascular and the complement-mediated inflammation might lead to retinal damages [51–53]. As reported from evidence in the literature, significant correlations occur between OCT-A abnormalities and disease activity in systemic autoimmune diseases as SLE suggesting that retinal capillary plexi may represent the “sentinel” of the microvascular network involved in systemic inflammatory disorders [29–33]. Accordingly, we documented in our AAV cohort that disease damage resulted directly related with the BVAS-disease activity as well as the disease duration: interestingly, vessel density from AAV patients resulted negatively related with disease-related damage (assessed by VDI) suggesting that a higher disease activity a greater microvascular injury. BVAS is a comprehensive multisystem clinical assessment in AAV and includes eyes in terms of scleritis/episcleritis, conjunctivitis/blepharitis/keratitis, and uveitis. Also, retinal changes are analyzed in BVAS as vasculitis, thrombosis, exudate, hemorrhages: however, all these abnormalities in pre-symptomatic phases can be undiagnosed and, thus, can lead to subclinical chronic damages, at the same time [22, 25, 26]. In accordance with our findings, the OCT-A can be considered as a key diagnostic tool in the early detection of possible subclinical retinal changes in AAV by adding information on disease-damage and activity in pre-symptomatic patients.

Non-specific microvascular abnormalities have been described by NVC in most AAV patients, with no correlation with disease activity and damage. Accordingly, a recent study reported NVC-scleroderma patterns only in a small percentage of AAV patients [38]. Thus, preliminary data suggest that AAV patients present microvascular abnormalities at NVC, whose clinical relevance certainly requires further studies [38]. Moreover, in accordance with the hypothesis of a potential agreement between microvascular changes at retinal level and at nailfold bed, we analyzed both OCT-A and NVC measures and no relevant correlation resulted.

Main limitation of these findings is the sample size of the AAV cohort: a larger population is needed to better stratify patients based on AAV type and, thus, clinical phenotype, comorbidities, and concomitant therapies. In addition, there are also risks for selection bias including the fact that the study cohort comprised highly selected subjects without comorbidities, which is a rare condition in AAV patients. Furthermore, included AAV patients showed a mild disease, in a significant proportion without disease modifying treatments. Therefore, how applicable the present findings are in the real practice could be a challenge.

## Conclusions

Our results may represent the first hypothesis-generating basis for defining the role of OCT-A in non-symptomatic AAV patients to obtain an early diagnosis of retinal involvement, an accurate detection of disease-damage, and a more tailored treatment and management of such rare and complexes patients. In AAV patients, the role of NVC to define microvascular abnormalities requires further studies.

**Table 1:** Data from the study population

	AAV (n = 23)	HC (n = 20)
GPA (N/%)	5/22	N/A
EGPA (N/%)	9/39	N/A
MPA (N/%)	9/39	N/A
Age at the study (mean ± SD)	60.9 ± 8.7	57.9 ± 10.7
M:F	0.8	1
Disease duration (yrs, mean ± SD)	9.6 ± 9.1	N/A
**Clinical Features**		
ENT	15/65	N/A
Kidney	4/17	N/A
Heart	4/17	N/A
Lung	21/91	N/A
Skin	6/26	N/A
Joint	9/39	N/A
Myalgia	7/30	N/A
PNS	13/57	N/A
Gut	3/13	N/A
**Laboratory Assays**		
C3 (mg/dl)	125.4 ± 20.8	N/A
C4 (mg/dl)	27.3 ± 9.7	N/A
Elevated IgE (> 90 UI/ml)	3/13	N/A
c-ANCA (≥ 2,3 UI/ml)	5/21.8	N/A
p-ANCA (≥ 3,2 UI/ml)	8/34.8	N/A
glucose (mg/dl)	80.7 ± 17.3	N/A
creatinine (mg/dl)	0.95 ± 0.3	N/A
24- PTU (mg/24 h)	122.4 ± 62.8	N/A
BVAS	3.3 ± 2.5	N/A
VDI	4.5 ± 1.5	N/A
FFS	0.3 ± 0.4	N/A
**Treatments**		
Steroids	18/78	N/A
Hydroxycloroquine	1/4	N/A
c/b-DMARDs	17/74	N/A

Abbreviation: AAV, Anti-neutrophil cytoplasm autoantibodies (ANCA)-associated vasculitides; HC, healthy controls; GPA, granulomatosis with polyangiitis; EGPA, Eosinophilic granulomatosis with polyangiitis; MPA, microscopic polyangiitis; N/A, not applicable; SD, standard deviation; M, male; F, female; yrs, years; PNS, peripheral nervous system; C3/C4, complement; Ig, Immunoglobulin; p-ANCA, perinuclear-ANCA; c-ANCA, cytoplasmatic-ANCA; 24-PTU, 24 hour proteinuria; BVAS, Birmingham Vasculitis Activity Score; VDI, Vasculitis Damage Index; FFS, Five Factor Score; c/bDMARDs, conventional synthetic/ biological disease-modifying antirheumatic drugs. Continuous variables are shown as means ± SD, while categorical variables are absolute frequencies and percentages. Continuous variables were compared using the parametric unpaired t-test. Categorical variables were compared using the Chi-squared test or Fisher’ exact test when appropriate

**Table 2:** Retinal vessel density by optical coherence tomography angiography (OCT-A)

OCT-A	AAV (R) (n = 23)	AAV (L) (n = 23)	AVV (B) (n = 46)	HC (R) (n = 20)	HC (L) (n = 20)	HC (B) (n = 40)
SWD mean ± SD	49.5 ± 3.3 ^*^	50.5 ± 3.8 ^*^	50 ± 3.7 ^*^	52.4 ± 2.9	51.7 ± 3.6	52 ± 3.3
SWD min-max	41.6–53.8 ^*^	42.9–56.6^*^	41.6–56.6 ^*^	46.6–56.9	45.9–57	45–57
SPFD mean ± SD	51.7 ± 5.3 ^*^	52.9 ± 5 ^*^	52.3 ± 5.3 ^*^	55.6 ± 2.8	54.4 ± 3.9	55 ± 3.4
SPFD min-max	37.8–58.2^*^	40.3–61.3^*^	37.8–61.3 ^*^	48–59.1	47.4–60	47.4–60.6
DWD mean ± SD	51.5 ± 8^****^	51.7 ± 7.2 ^****^	51.6 ± 7.6^****^	58.4 ± 3.4	58.6 ± 3.6	58.5 ± 3.5
DWD min-max	36.1–63.5 ^****^	39.8–65.5 ^****^	36.1–65.5 ^****^	47.7–62.2	49.6–63.2	47.7–63.2
DPFD mean ± SD	56.2 ± 4.7^****^	56 ± 4.5^****^	56.1 ± 4.7^****^	61.4 ± 2.8	61.3 ± 4.2	61.4 ± 3.5
DPFD min-max	47.5–63.3 ^****^	47.8–63.9 ^****^	47.5–63.9 ^****^	53.3–64	51.2–66.6	51.2–66.6
FAZ mean ± SD	0.32 ± 0.23	0.27 ± 0.1	0.28 ± 0.1	0.23 ± 0.1	0.23 ± 0.1	0.23 ± 0.09

Abbreviation: OCT-A, optical coherence tomography angiography; AAV, Anti-neutrophil cytoplasm autoantibodies (ANCA)-vasculitides; HC, healthy controls; R, right; L, left; B, both; SWD, superficial whole density; SPFD, superficial parafoveal density; DWD, deep whole density; DPFD, deep parafoveal density; FAZ, Foveal avascular zone. Continuous variables are shown as means ± SD and range min-max. ^*^ AAV vs. HC (^*^ p < 0.05, ^****^ p < 0.0001)

**Table 3:** Retinal thickness by optical coherence tomography (OCT) scans

	AAV (R) (n = 23)	AAV (L) (n = 23)	AVV (B) (n = 46)	HC (R) (n = 20)	HC (L) (n = 20)	HC (B) (n = 40)
FT (µm) mean ± SD	254.6 ± 20.3	254.5 ± 20.5	254.5 ± 20	258 ± 17.5	262.6 ± 20.9	260 ± 19
FT (µm) min-max	221–293	223–302	221–302	237–294	220-296t	220–296
PFT (µm) mean ± SD	324.6 ± 14	323.6 ± 12.8	324 ± 13.2	319 ± 13.8	322.6 ± 10.5	320.9 ± 12.3
PFT (µm) min-max	301–341	304 − 242	301–342	297–351	299–344	297–351

Abbreviation: AAV, Anti-neutrophil cytoplasm autoantibodies (ANCA)-vasculitides; HC, healthy controls; R, right; L, left; B, both; FT: Foveal Thickness; PFT: Parafoveal thickness. Continuous variables are shown as means ± SD and range min-max

## Data Availability

materials. The data presented in this study are available on request from the corresponding author.

## Abbreviations

AAV: ANCA-associated vasculitides
ANA: anti-nuclear antibodies
ANCA: anti-neutrophil cytoplasm autoantibodies
c-ANCA: cytoplasmatic ANCA
p-ANCA: perinuclear ANCA
BCVA: best-corrected visual acuity
BVAS: Birmingham Vasculitis Activity Score
C3: complement components C3
C4: complement components C4
CLIA: chemiluminescent Immunoassay
CSS: Churg-Strauss Syndrome
DPFD: deep parafoveal vessel density
DWD: deep whole vessel density
EGPA: eosinophilic granulomatosis with polyangiitis
ENT: ear-nose-throat
ETDRS: Early Treatment of Diabetic Retinopathy Study
FAZ: Foveal avascular zone
FFS: Five Factor Score
GPA: granulomatosis with polyangiitis
HC: healthy controls
IFA: immunofluorescence assay
***Ig***: Immunoglobulin
IOP: intraocular pressure
MPA: microscopic polyangiitis
MPO: myeloperoxidase
NVC: nailfold video-capillaroscopy
OCT-A: optical coherence tomography angiography
PNS: peripheral nervous system
PR3: proteinase 3
RF: rheumatoid factor
SLE: Systemic Lupus Erythematosus
SPFD: superficial parafoveal vessel density
SWD: superficial whole vessel density
VD: vessel density
VDI: Vasculitis Damage Index
